# Supplementary material for: Reverse Osmosis with Intermediate Chemical Demineralization: Scale Inhibitor Selection, Degradation, and Seeded Precipitation
Source: Molecules. 2024 May 7;29(10):2163. doi: 10.3390/molecules29102163 (PMC11124285; doi:10.3390/molecules29102163)
Supplement: Supplementary file 1 [file molecules-29-02163-s001.zip › molecules-2962392-supplementary.pdf]

Support Information

Reverse Osmosis with Intermediate Chemical Demineralization: Scale Inhibitor Selection, Degradation and Seeded Precipitation

Shichang Xu [1], Ping Wang [1], Lixin Xie [1,*], Yawei Du [2] and Wen Zhang [1,*]

1 Tianjin Key Laboratory of Membrane Science and Desalination Technology, State Key Laboratory of Chemical Engineering, School of Chemical Engineering and Technology, Tianjin University, Tianjin 300350, China; xu_sc1@tju.edu.cn (S.X.); pingwang_971213@tju.edu.cn (P.W.)

2 School of Chemical Engineering and Technology, Hebei University of Technology, Tianjin 300401, China; sonicduyawei001@126.com

* Correspondence: xie_lixin@tju.edu.cn (L.X.); zhang_wen@tju.edu.cn (W.Z.)

Table number 2, Figure number 3

Table S1 Ratio of residual scale inhibitor (R) after the UVD stage for different operating conditions

Exp. No.	Scale inhibitor	UV intensit	H_2O_2 (mg/L)	t_p (mi	Loading mount	Seeds particle	*R*
PBL	-	-	-	-	2.5	7-43	0
NBL	10	-	-	-	2.5	7-43	1
(1)	10	20	70	15	2.5	7-43	0.03
	10	30	70	15	2.5	7-43	0.02
	10	40	70	15	2.5	7-43	0
(2)	10	40	70	15	2.5	7-43	0
	10	40	40	15	2.5	7-43	0.01
	10	40	10	15	2.5	7-43	0.07
(3)	10	20	70	15	2.5	7-43	0.03
	10	20	70	20	2.5	7-43	0
	10	20	70	25	2.5	7-43	0
	10	20	70	30	2.5	7-43	0
(4)	10	20	70	15	2.5	7-43	0.03
	10	20	70	15	2.5	43-71	0.03
	10	20	70	15	2.5	71-91	0.03
(5)	10	20	70	15	1.5	7-43	0.03
	10	20	70	15	2.5	7-43	0.03
	10	20	70	15	3.5	7-43	0.03
	10	20	70	15	5.5	7-43	0.03
(6)	10	40	70	15	2.5	7-43	0
	10	40	70	15	2.5	from	0
	10	40	70	15	2.5	from H2	0
	10	40	70	15	2.5	from H3	0

Table S2 Composition of model concentrates with different gypsum saturation indices

[a] K stands for the concentration ratio.

salt	$CaCl_2$ (mmol/L)	Na_2SO_4 (mmol/L)	NaCl (mmol/L)	pH	SIg
PRO concentrate model solution(A)	12.85	20.7466	26.4016	6.70	0.70
K[a]=1.5	19.27	31.12	39.60	6.70	1.16
K=2.0	25.69	41.49	52.80	6.70	1.66
K=2.5	32.11	51.87	66.00	6.70	2.19
K=3.0	38.54	62.24	79.20	6.70	2.75
K=3.5	44.96	72.61	92.41	6.70	3.34
K=4.0	51.38	82.99	105.61	6.70	3.94
K=4.5	57.80	93.36	118.81	6.70	4.57
K=5.0	64.23	103.73	132.00	6.70	5.21
K=5.5	70.65	114.10	145.21	6.70	5.87
K=6.0	77.07	124.48	158.41	6.70	6.54
K=6.5	83.49	134.85	171.61	6.70	7.23

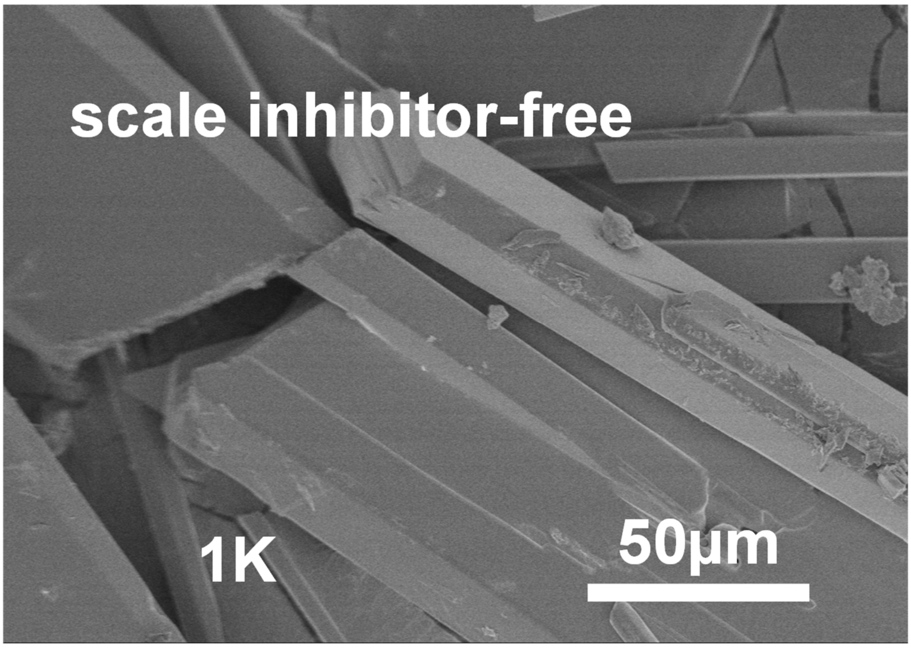

Figure S1 SEM images of scaling materials after 10h without scale inhibitor (SIg=6.5).

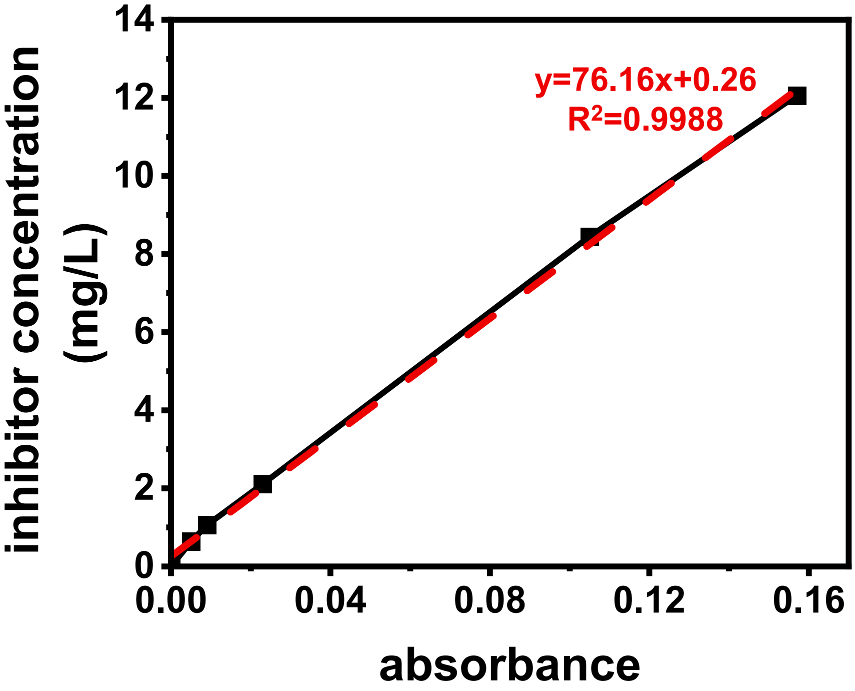

Figure S2 The standard curve of RO-400 concentration.

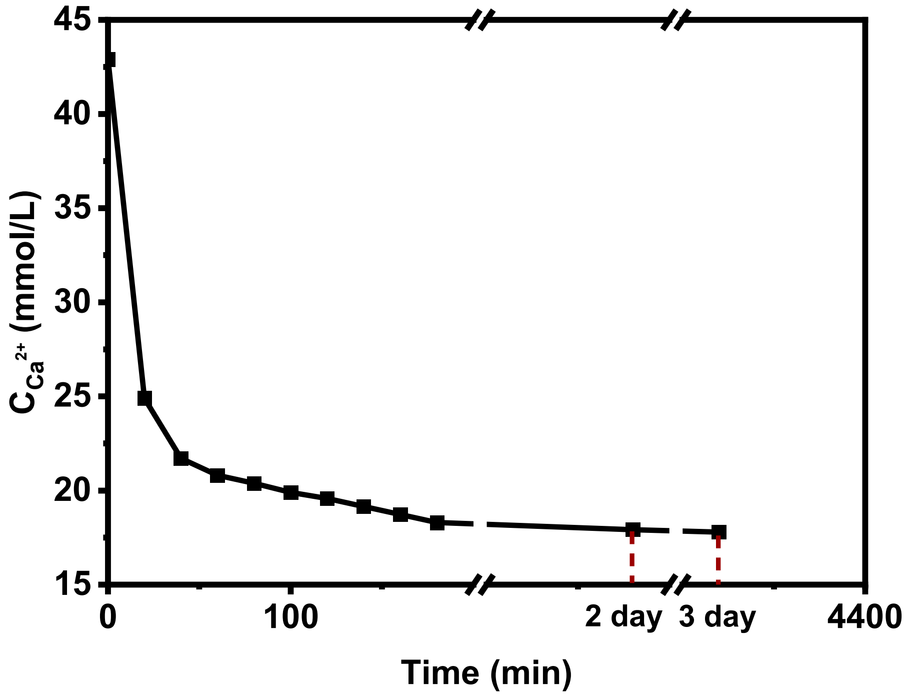

Figure S3 Scale inhibitor-free calcium sulfate model solution C-bulk actual precipitation process ($c_{3\ day}$ considered as c_{eq})
